# *In Vivo* Mutational Characterization of DndE Involved in DNA Phosphorothioate Modification

**DOI:** 10.1371/journal.pone.0107981

**Published:** 2014-09-30

**Authors:** Chongde Lai, Xiaolin Wu, Chao Chen, Teng Huang, Xiaolin Xiong, Shuangju Wu, Meijia Gu, Zixin Deng, Xi Chen, Shi Chen, Lianrong Wang

**Affiliations:** 1 Key Laboratory of Combinatorial Biosynthesis and Drug Discovery, Ministry of Education, and School of Pharmaceutical Sciences, Wuhan University, Wuhan, China; 2 Taihe Hospital, Hubei University of Medicine, Shiyan, Hubei, China; 3 College of Bioscience and Bioengineering, Jiangxi Agricultural University, Nanchang, China; New England Biolabs, Inc., United States of America

## Abstract

DNA phosphorothioate (PT) modification is a recently identified epigenetic modification that occurs in the sugar-phosphate backbone of prokaryotic DNA. Previous studies have demonstrated that DNA PT modification is governed by the five DndABCDE proteins in a sequence-selective and *R*_P_ stereo-specific manner. Bacteria may have acquired this physiological modification along with *dndFGH* as a restriction-modification system. However, little is known about the biological function of Dnd proteins, especially the smallest protein, DndE, in the PT modification pathway. DndE was reported to be a DNA-binding protein with a preference for nicked dsDNA *in vitro*; the binding of DndE to DNA occurs via six positively charged lysine residues on its surface. The substitution of these key lysine residues significantly decreased the DNA binding affinities of DndE proteins to undetectable levels. In this study, we conducted site-directed mutagenesis of *dndE* on a plasmid and measured DNA PT modifications under physiological conditions by mass spectrometry. We observed distinctive differences from the *in vitro* binding assays. Several mutants with lysine residues mutated to alanine decreased the total frequency of PT modifications, but none of the mutants completely eliminated PT modification. Our results suggest that the nicked dsDNA-binding capacity of DndE may not be crucial for PT modification and/or that DndE may have other biological functions in addition to binding to dsDNA.

## Introduction

Phosphorothioate linkage was originally developed as an artificial means to stabilize oligonucleotides against nuclease degradation [1]. However, physiological DNA PT modification was recently discovered as a new type of epigenetic modification in which the nonbridging oxygen is replaced with sulfur [2]. Previous studies have shown that PT modification is governed by the products of the five clustered *dndABCDE* genes [3]. PT linkages are susceptible to Tris peroxide, which accumulates on the anode during conventional or pulsed-field gel electrophoresis, resulting in DNA degradation [3], [4]. DNA PT modification was first discovered in *Streptomyces lividans*, and it was later found to be widespread across a range of species due to the horizontal transfer of the *dnd* genes [3], [5], [6], [7]. The genomic mapping of PT sites across bacterial genomes revealed short consensus sequences and partial modifications at given sites [8]. Moreover, a three-gene cluster, *dndFGH*, which is located adjacent to the *dndBCDE* in approximately 86 bacterial species constitutes a restriction-modification system to restrict foreign DNA. This system displays similarities to methylation-based R-M systems [9], [10], [11], [12]. However, more than 100 strains possess only *dndBCDE* and lack *dndFGH*, suggesting that these genes may encode proteins with functions other than those of a typical R-M system.

An emerging model of Dnd protein functions hypothesizes that DndA is a cysteine desulfurase that catalyzes the removal of sulfur from L-cysteine and assembles DndC as a 4Fe-4S cluster protein. DndC is predicted to have PAPS reductase activity and possesses ATP pyrophosphatase activity [13]. DndB shows homology to a group of transcriptional regulators, whereas DndD has ATPase activity that is potentially related to DNA nicking during sulfur incorporation [3], [14]. DndE is the smallest Dnd protein and consists of 117 amino acids in both *Salmonella enterica* serovar Cerro 87 and *Escherichia coli* B7A. It is believed that the DNA phosphodiester linkage within the consensus sequence is nicked by DndD prior to sulfur replacement [14].

DndE is a tetrameric protein that shows a stronger binding affinity for dsDNA with a phosphorylated nick than for intact dsDNA [15]. Six positively charged lysine residues on the surface of DndE are predicted to be involved in the selective binding to negatively charged phosphorylated gaps. Hu *et al.* replaced the positively charged lysine residues on the DndE surface (K17, K18, K20, K53, K87 and K91) with alanine individually and then tested by binding affinity of the DndE mutants for DNA substrates *in vitro*. The DndE variants DndE_K17A_, DndE_K20A_, DndE_K53A_ and DndE_K91A_ showed 3- to 8-fold decreased binding affinities to intact dsDNA and displayed non-detectable binding to nicked dsDNA [15]. In particular, the mutagenesis of the K18 residue reduced the binding of DndE to both intact and nicked dsDNA below detectable levels [15].

To investigate the impact of the key lysine residues on DNA PT modifications under physiological conditions, we conducted site-directed mutagenesis of *dndE* in the plasmid pJTU1238, which contains the *dndBCDE* cluster from *S. enterica* and confers PT-modified d(G_PS_A) and d(G_PS_T) to *E. coli* hosts [6]. The plasmid pJTU1238 and its derivatives were then transformed into *E. coli* DH10B and XTG102, the *S. enterica dndBCDE* deletion mutant. The abundance of DNA PT modifications was measured by liquid chromatography coupled with tandem mass spectrometry (LC-MS/MS). We found that mutating the individual lysines in DndE altered the DNA PT modifications in several ways. However, none of the mutations completely blocked the PT modification pathway. Our results reveal that the disruption of the nicked dsDNA-binding activity of DndE may not be sufficient to abolish DNA PT modifications. Meanwhile, DndE may not be uniquely designed to interact with nicked dsDNA, and therefore may have other functions.

## Materials and Methods

### Bacterial strains, plasmids and primers

The bacterial strains, plasmids, and primers used in this study are listed in Table 1.

**Table 1:** Strains, plasmids and primers used in this study.

Strains	Characteristics	Reference
*E. coli* DH10B	*E. coli* host for pJTU1238 and derivatives	Invitrogen [17]
XTG102	*S. enterica* derivative, *dndBCDE* deletion mutant	[9]

Plasmids	Characteristics	Reference
pBluescript II SK(+)	Cloning vector,Amp^r^	[18]
pJTU1238	*dndB-E* from *S. enterica*, cloned in pBluescript SK+	[2]
pWHU669	pJTU1238 derivative site mutant with K17A	This study
pWHU670	pJTU1238 derivative site mutant with K18A	This study
pWHU671	pJTU1238 derivative site mutant with K18A and K20A	This study
pWHU672	pJTU1238 derivative site mutant with K53A	This study
pWHU673	pJTU1238 derivative site mutant with K87A	This study
pWHU674	pJTU1238 derivative site mutant with K91A	This study

Primers	Sequences	Use
K17A F	CAGACTGAAGACCAGCTTGCAAAGCTAAAAGG	This study
K17A R	CCTTTTAGCTTTGCAAGCTGGTCTTCAGTCTG	This study
K18A F	GAAGACCAGCTTAAAGCGCTAAAAGGATATACG	This study
K18A R	CGTATATCCTTTTAGCGCTTTAAGCTGGTCTTC	This study
K20A F	CAGCTTAAAAAGCTAGCAGGATATACGGGGATT	This study
K20A R	AATCCCCGTATATCCTGCTAGCTTTTTAAGCTG	This study
K18A-K20A F	GACCAGCTTAAAGCGCTAGCAGGATATACGGGG	This study
K18A-K20A R	CCCCGTATATCCTGCTAGCGCTTTAAGCTGGTC	This study
K53A F	CGGGATAGTAAGGCGCTGGATGGCTCT	This study
K53A R	AGAGCCATCCAGCGCCTTACTATCCCG	This study
K87A F	CCGCAGCTAGAGCAGGCAATACTAATTAAAGCA	This study
K87A R	TGCTTTAATTAGTATCGTCTGCTCTAGCTGCGG	This study
K91A F	AAAATACTAATTGCAGCATGGGCAGCA	This study
K91A R	TGCTGCCCATGCCGTAATTAGTATTTT	This study
Δ*dndE*-1	GACAGGAATGCGATGGAT	This study
Δ*dndE*-2	CCTTAATGCAGCAATTCCATCTTCAGTCTGACGGCTAAGT	This study
Δ*dndE*-3	ACTTAGCCGTCAGACTGAAGATGGAATTGCTGCATTAAGG	This study
Δ*dndE*-4	CGGTGATGTCGGCGATAT	This study
*dndE*-Mu-F	CGATACTGAAGTGGACGAG	This study
*dndE*-Mu-R	AAGACCAAACTATAATTTTCG	This study

### The in-frame deletion of *dndE* in pJTU1238

**Figure 1:**
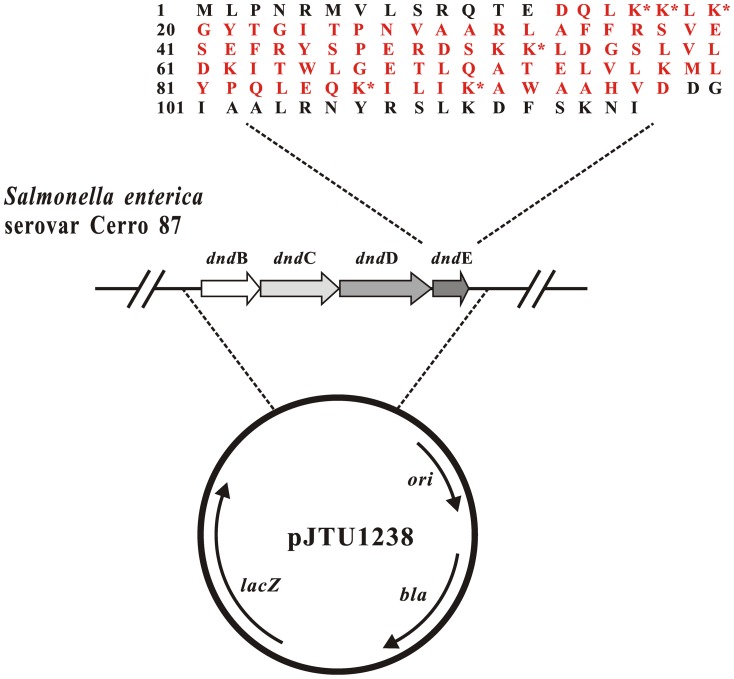
Site-directed mutagenesis of the lysine residues of the *dndE* gene in pJTU1238. The indicated mutations of key lysine residues (K17, K18, K20, K53, K87 and K91) were constructed in pJTU1238, a plasmid harboring the *dndBCDE* genes of *S. enterica*. The asterisks denote the selected lysine residues that were replaced with alanines. The residues marked in red indicate the amino acids that are deleted in the *dndE* in-frame deletion mutant.

The in-frame deletion of *dndE* in pJTU1238 was constructed by two-step PCR. In the first step, the upstream and downstream fragments of *dndE* were generated by PCR using the primer pairs Δ*dndE*-1/Δ*dndE*-2 and Δ*dndE*-3/Δ*dndE*-4, respectively (Table 1). Δ*dndE*-2 and Δ*dndE*-3 are chimeric primers. A mixture of two purified PCR products, which overlapped by 40 bp, served as the template for a ligation PCR using primers Δ*dndE*-1 and Δ*dndE*-4. An 1,877-bp product containing the *dndE* deletion fragment was generated and cloned into the pEASY-Blunt Zero vector (TransGen Biotech), yielding plasmid pWHU675. This plasmid was confirmed by sequencing analysis. The NsiI-XhoI fragment of pWHU675 containing an in-frame deletion of *dndE* (corresponding to amino acid nos. 14 to 98; red in Figure 1) was inserted into the corresponding sites in pJTU1238 to generate the plasmid pWHU676.

### Site-directed mutagenesis of *dndE*

The site-directed mutagenesis of the *dndE* gene on plasmid pJTU1238 was performed using the Muta-direct Site Directed Mutagenesis Kit (SBS Genetech). The primers were designed according to the manufacturer's instructions. The six positively charged lysine residues on the DndE surface (K17, K18, K20, K53, K87 and K91) were individually replaced with alanine (Figure 1). The mutations on pJTU1238 were verified by DNA sequencing using the primers *dndE*-Mu-F and *dndE*-Mu-R (Table 1). The derived plasmids were then transformed into XTG102, a *S. enteric*a mutant strain with a deletion of *dndBCDE*, and *E. coli* DH10B to detect PT modifications by LC-MS/MS.

### DNA digestion conditions

All strains were grown in LB medium to an OD600 of 0.8 at 28°C for *Salmonella* and 37°C for *E. coli*. DNA was isolated using the QIAGEN Genomic-tip 100/G kit. After isolation, 20 µg of DNA was hydrolyzed with 2 U of nuclease P1 (USBiological) in 30 mM sodium acetate, pH 5.3, and 0.5 mM ZnCl_2_ in a total volume of 100 µL at 50°C for 2 h. Following the addition of 10 µL of 1 M Tris-Cl (pH 8.0) to adjust the pH, dephosphorylation was performed by 4 U of alkaline phosphatase (Sigma) at 37°C for another 4 h. The enzymes were subsequently removed by passing the reaction mixture over a PALL Nanosep 10 K OMEGA centrifugal column, and the reaction was concentrated under a vacuum. Prior to the quantitative analysis, the external calibration curves containing varying amounts of d(G_PS_A) *R*_P_ and d(G_PS_T) *R*_P_ standards (5, 10, 20, 40, 60, and 80 pmol) were plotted with 20 pmol of d(G_PS_A) *S*_P_ as a reference. The correlation coefficients (r2) were greater than 0.999.

### LC-MS/MS analysis of DNA PT modifications

The digested DNA samples were resolved by a Thermo Hypersil GOLD aQ column (150×2.1 mm, 3 µm) by elution at 35°C. The flow rate was set to 200 µL/min using a gradient starting at 97% buffer A (0.1% acetic acid in water) and 3% buffer B (0.1% acetic acid in acetonitrile) for 5 min, followed by a linear gradient to 6% buffer B over 30 min and 6% buffer B to 98% buffer B over 1 min. The buffer was held at 98% buffer B for another 15 min and then changed back to the starting concentration of 3% buffer B over 1 min, followed by a 15 min period to allow the column to re-equilibrate to the 3% buffer B. The column was coupled to a Thermo TSQ Quantum Access MAX mass spectrometer with an electrospray ionization source in positive mode. The selected reaction-monitoring (SRM) scan mode in MS/MS was employed to detect the following ions: d(G_PS_A) *R*_P_ [M + H]+ *m/z* 597 → 136, d(G_PS_T) *R*_P_, [M + H]+ *m/z* 588 → 152 and d(G_PS_A) *S*_P_ [M + H]+ *m/z* 597 → 136. The following parameters were optimized for maximal sensitivity: gas flow, 10 L/min; nebulizer pressure, 25 psi; drying gas temperature, 300°C; capillary temperature, 320°C; source spray voltage, 3.5 kV; sheath gas setting, 40; aux gas setting, 8; capillary voltage, 35 V; and tube lens voltage, 110 V. The collision energy and collision gas pressure were chosen to achieve the lowest background noise.

### Quantitative RT-PCR

To assess the expression levels of the *dndE* genes, total RNA was isolated using a Qiagen RNeasy Protect Bacteria Mini Kit. Samples of 1 µg total RNA were reverse transcribed to synthesize cDNA using RevertAid First Stand cDNA Synthesis kits (Thermo Scientific). Then, 20 ng of cDNA was used as a template for qualitative real-time PCR performed with the SsoFast EvaGreen Supermix with Low ROX Kit (Bio-Rad) and a 7900 HT Fast Real-Time PCR System (Applied Biosystems). An initial incubation at 95°C for 30 s was followed by 40 cycles of 95°C for 10 s and 58.5°C for 30 s. The housekeeping gene *gapA*, which encodes D-GAPDH, was used as a reference. The primers 5′- TGCTCCCGAATCGAATGGTA-3′ and 5′-AGCGAAACTCACTCTCCACT-3′ were used to amplify *dndE*, primers 5′-GAAGGCCAGGACATCGTTTC-3′ and 5′-AGTCGCGTGAACAGTAGTCA-3′ were used to amplify *gapA* from XTG102, and primers 5′-CACGCTACTACCGCTACTCA -3′ and 5′- AGGACGGGATGATGTTCTGG-3′ were used to amplify *gapA* from *E. coli* DH10B. The RT-PCR data analysis was performed according to the comparative threshold cycle method (also known as 2^−ΔΔCT^) between the different strains.

## Results

### Expression of pJTU1238 and its derivatives in *E. coli*

We previously reported that the plasmid pJTU1238 containing the *dndBCDE* gene cluster conferred the PT modifications of d(G_PS_A) *R*_P_ and d(G_PS_T) *R*_P_ to *E. coli* DH10B [6]. As a high copy number plasmid, pJTU1238 increased the expression of the *dndBCDE* genes, leading to a 2-fold increase in the total number of PT modifications compared to wild-type *S. enterica* [6]. The in-frame deletion of *dndE* in pJTU1238, however, completely abolished DNA PT modifications, suggesting that *dndE* plays an essential role in the modification pathway (Figure 1; Table 2). To evaluate the influence of the positively charged lysine residues on DNA PT modification under physiological conditions, mutations were made in pJTU1238 to generate a set of plasmids, pWHU668 to pWHU674, carrying the *dnd* gene cluster and encoding DndE derivatives (Table 1). The individual replacement of each lysine residue with alanine did not alter the PT sequence specificity of d(G_PS_A) and d(G_PS_T) (Table 2). The DNA PT frequencies were normalized to *dndE* transcript levels, and we found that the individual K17A, K20A, K53A and K87A mutations decreased the total PT by 22% to 44%. The K53A mutation slightly increased the frequency of PT modifications by 10%, while the K91A mutation slightly decreased the total frequency of PT modifications in *E. coli* DH10B by 11%. None of the mutations resulted in a complete loss of PT modifications (Table 2 & 3).

**Table 2:** PT modifications in *E. coli* DH10B harboring pJTU1238 and its derivatives.

Mutations	Strains	PT modifications per 10^6^ nt		
		d(G_PS_A)	d(G_PS_T)	Total PT
wild type	DH10B(pJTU1238)	778±29	727±60	1505±89
K17A	DH10B(pWHU668)	662±34	624±27	1286±60
K18A	DH10B(pWHU669)	629±13	579±25	1208±31
K20A	DH10B(pWHU670)	720±12	647±5	1367±16
K53A	DH10B(pWHU672)	821±12	780±35	1601±46
K87A	DH10B(pWHU673)	765±10	718±17	1483±28
K91A	DH10B(pWHU674)	818±17	744±29	1562±43
K18A+K20A	DH10B(pWHU671)	569±8	510±4	1080±8
Δ*dndE*	DH10B(pWHU676)	ND	ND	ND

Values represent the mean ± SD for biological triplicates; ND, not detected.

**Table 3:** Total PT modifications normalized to *dndE* transcript levels.

Mutations	Strains	Normalized PT (%)	Strains	Normalized PT (%)
wild-type	DH10B(pJTU1238)	100	XTG102 (pJTU1238)	100
K17A	DH10B(pWHU668)	78.4	XTG102 (pWHU668)	65.3
K18A	DH10B(pWHU669)	110.0	XTG102 (pWHU669)	45.9
K20A	DH10B(pWHU670)	55.8	XTG102 (pWHU670)	57.6
K53A	DH10B(pWHU672)	65.4	XTG102 (pWHU672)	58.9
K87A	DH10B(pWHU673)	60.1	XTG102 (pWHU673)	69.6
K91A	DH10B(pWHU674)	89.5	XTG102 (pWHU674)	103.5
K18A+K20A	DH10B(pWHU671)	33.2	XTG102 (pWHU671)	38.3
Δ*dndE*	DH10B(pWHU676)	ND	XTG102 (pWHU676)	ND

ND, not detected.

### Expression of pJTU1238 and derivatives in *S. enterica* mutant

**Figure 2:**
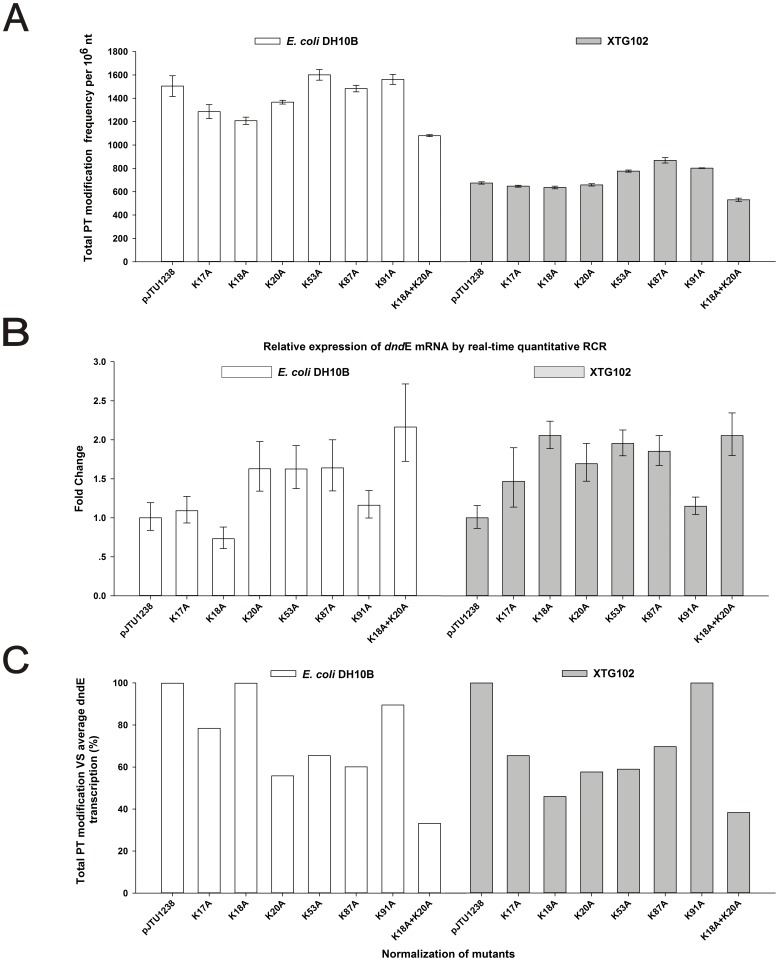
The impact of the mutagenesis of the lysine residues on the total PT modifications. A. The plasmids pWHU668-pWHU674 possessing mutated lysine residues were expressed in *E. coli* DH10B and XTG102, and the DNA PT frequencies were measured by LC-MS/MS. The columns and error bars represent the mean ± SD; n = 3, biological triplicates. B. qRT-PCR expression analysis of the *dndE* genes in *E. coli* DH10B (white bars) and XTG102 (grey bars). The expression levels were normalized to the transcript levels of GAPDH. The columns and error bars represent the mean ± SD; n = 3, biological triplicates. C. PT modifications were normalized to the average *dndE* expression levels.

To validate the role of mutated DndE in the native host, pJTU1238 and its derivatives were transformed into XTG102, the *dndBCDE* in-frame deletion mutant of *S. enterica* (Table 3 & 4). The K17A, K18A, K20A, K53A and K87A mutations in XTG102 reduced the normalized PT by 30% to 54%, while K91A slightly increased the number of PT modifications by 4%. As shown in Figure 2, no lysine replacement completely abolished PT modification. We previously reported that the overexpression of *dndBCDE* on the high-copy pBluescript SK+ plasmid or the low-copy pACYC184 plasmid caused PT modifications to increase by 2- and 1.5-fold, respectively [6]. However, the overexpression of the *dnd* cluster on pJTU1238 did not cause a significant increase in the total PT frequency in XTG102, suggesting that the frequency of DNA PT modification in *Salmonella* is regulated.

**Table 4:** PT modifications in XTG102 harboring pJTU1238 and its derivatives.

Mutations	Strains	PT modifications per 10^6^ nt		
		d(G_PS_A)	d(G_PS_T)	Total PT
wild type	XTG102(pJTU1238)	374±5	299±5	673±10
K17A	XTG102 (pWHU668)	383±3	264±5	646±8
K18A	XTG102 (pWHU669)	383±8	251±4	635±11
K20A	XTG102 (pWHU670)	392±4	265±12	657±10
K53A	XTG102 (pWHU672)	398±8	377±16	775±9
K87A	XTG102 (pWHU673)	451±11	417±14	868±24
K91A	XTG102 (pWHU674)	410±8	390±8	800±3
K18A+K20A	XTG102 (pWHU671)	297±5	233±9	530±14
Δ*dndE*	XTG102 (pWHU676)	ND	ND	ND

Values represent the mean ± SD for biological triplicates; ND, not detected.

### Double-site mutagenesis of *dndE*

X-ray crystallography analysis of DndE revealed that K20 was involved in the formation of hydrogen bonds that produce a positively charged hole [15]. The K20 residue was also proposed as one of the two key residues in DndE based on a DNA-binding affinity assay [15]. Here, we performed double-site mutagenesis, yielding the mutated plasmid pWHU671 encoding DndE with both K18 and K20 replaced with alanine residues. The double-site mutation led to the most significant decrease in total PT modifications, 67% and 62% in DH10B and XTG102, respectively. Although DndE_K18A_ and DndE_K20A_ displayed the most significant decreases in binding affinities to intact dsDNA (native DndE, K_D_ ∼88.3 µM; DndE_K20A_, K_D_ ∼690 µM; DndE_K18A_, non-detectable) and nicked dsDNA (DndE_K18A_ and DndE_K20A_, non-detectable) in the study by Hu *et al.* [15], the results of the present study showed that the double-site mutation had a remarkable effect on the Dnd modifying efficiency but did not inactivate the effect of the physiological DndE in the PT modification pathway.

## Discussion

There are five Dnd proteins involved in the biological pathway of sequence-selective and *R*p configuration-specific DNA PT modification. Protein-protein interactions were detected between DndA and DndC, as well as between DndA and DndE, indicating the formation of a Dnd complex [16]. The smallest Dnd protein, DndE, was previously believed to be a sulfotransferase or a phosphoribosylaminoimidazole carboxylase analogue by homology analysis [3]. However, the crystal structure proposed that DndE was a nicked dsDNA-binding protein. The six positively charged lysines on the DndE surface were speculated to be involved in the interaction with nicked dsDNA. The individual mutation of the six lysine residues was later found to significantly decrease the dsDNA-binding affinity *in vitro* [15]. The mutagenesis of pJTU1238 and the sensitive LC-MS/MS used in this study allowed us to monitor the physiological impact of these positively charged lysine residues on DNA PT modification.

In addition to the non-detectable affinities of DndE_K17A_, DndE_K18A_, DndE_K20A_, DndE_K53A_ and DndE_K91A_, the DndE_K87A_ mutation showed a decreased K_D_ of 323.5 µM in comparison to 30.6 µM of native DndE *in vitro* [15]. Our resulted showed that the mutations of K17A, K20A, K53A and K87A on pJTU1238 reduced the total PT frequencies in both *E. coli* DH10B and XTG102. However, none of the mutations abolished PT modification under physiological conditions. K18 and K20 were characterized as the two most essential lysine residues in binding nicked dsDNA *in vitro*. However, the K18 and K20 double mutant DndE protein encoded by pWHU671 was still able to generate PT modifications in *E. coli* DH10B and XTG102, albeit at significantly reduced levels of 33% and 38% compared to pJTU1238, respectively. Surprisingly, we observed an increased level of PT modification after mutating K18A in *E. coli* DH10B and K91A in XTG102. The comparison of DNA PT modifications *in vivo* and the DNA-binding affinity assay *in vitro* revealed that the disruption of the DNA-binding activity of DndE was not sufficient to abolish DNA PT modifications.

It is possible that *E. coli* may harbor unknown proteins having similar functions with *dndE*, which may complement the function of *dndE* when it is mutated. However, the complete loss of PT modification in the deletion mutant of *dndE* rules out this possibility. It is also possible that the mutation of lysine residues on DndE surface interfered the interaction of Dnd proteins to form complex, which resulted in the different efficiencies of DNA PT modification. Our findings suggest that the dsDNA-binding ability of DndE may not be crucial for its PT modification activity. Whether the perspective role of DndE is a sulfotransferase or a phosphoribosylaminoimidazole carboxylase remains to be validated. Our results also suggest that DndE may have other functions in the DNA PT modification pathway, and therefore may not be uniquely designed to interact with nicked dsDNA.
